# Full-Field Strain Uncertainties and Residuals at the Cartilage-Bone Interface in Unstained Tissues Using Propagation-Based Phase-Contrast XCT and Digital Volume Correlation

**DOI:** 10.3390/ma13112579

**Published:** 2020-06-05

**Authors:** Gianluca Tozzi, Marta Peña Fernández, Sarah Davis, Aikaterina Karali, Alexander Peter Kao, Gordon Blunn

**Affiliations:** 1 Zeiss Global Centre, School of Mechanical and Design Engineering, University of Portsmouth, Portsmouth PO1 3DJ, UK; martapf@kth.se (M.P.F.); katerina.karali@port.ac.uk (A.K.); alex.kao.port@gmail.com (A.P.K.); 2 School of Engineering Sciences, KTH Royal Institute of Technology, 100 44 Stockholm, Sweden; 3 School of Pharmacy and Biomedical Science, University of Portsmouth, Portsmouth PO1 2DT, UK; sarah.davis@port.ac.uk (S.D.); gordon.blunn@port.ac.uk (G.B.)

**Keywords:** X-ray computed tomography, phase-contrast imaging, cartilage-bone interface, digital volume correlation

## Abstract

A deeper understanding of the cartilage-bone mechanics is fundamental to unravel onset and progression of osteoarthritis, enabling better diagnosis and treatment. The aim of this study is therefore to explore the capability of X-ray computed (XCT) phase-contrast imaging in a lab-based system to enable digital volume correlation (DVC) measurements of unstained cartilage-bone plugs from healthy adult bovines. DVC strain uncertainties were computed for both articular cartilage and mineralized tissue (calcified cartilage and subchondral bone) in the specimens at increasing propagation distances, ranging from absorption up to four times (4× such effective distance. In addition, a process of dehydration and rehydration was proposed to improve feature recognition in XCT of articular cartilage and mechanical properties of this tissue during the process were assessed via micromechanical probing (indentation), which was also used to determine the effect of long X-ray exposure. Finally, full-field strain from DVC was computed to quantify residual strain distribution at the cartilage-bone interface following unconfined compression test (ex situ). It was found that enhanced gray-scale feature recognition at the cartilage-bone interface was achieved using phase-contrast, resulting in reduced DVC strain uncertainties compared to absorption. Residual strains up to ~7000 µε in the articular cartilage were transferred to subchondral bone via the calcified cartilage and micromechanics revealed the predominant effect of long phase-contrast X-ray exposure in reducing both stiffness and hardness of the articular cartilage. The results of this study will pave the way for further development and refinement of the techniques, improving XCT-based strain measurements in cartilage-bone and other soft-hard tissue interfaces.

## 1. Introduction

Osteoarthritis (OA) is one of the most prevalent and disabling chronic degenerative diseases with a 7-fold increase in its incidence predicted by 2030 [1]. It has been speculated that the osteoarthritic process may be triggered by an increase of thickness, volume and stiffness in the subchondral bone [2] due to repetitive joint loading that causes an initial increase in bone remodeling, perhaps as an adaptation to repair damage caused by such loading [3] A deep understanding of the cartilage-bone mechanics is therefore vital to explain load-transfer within the osteochondral tissue. So far, experimental strains in articular cartilage were mainly limited to two-dimensional (2D) image analysis (i.e., via digital image correlation (DIC) [4,5]) of local regions of the tissue, where their full interplay and extent in the cartilage-bone unit could not be interrogated. Digital volume correlation (DVC) using contrast-enhanced high-resolution X-ray computed tomography (XCT), where both the soft and calcified tissues can be visualized, would be able to capture the three-dimensional (3D) full-field strain distribution at the cartilage-bone interface over applied loads.

XCT-based DVC has been previously used to assess strain evolution in bone [6,7] and bone-biomaterial systems [8,9] under different loading conditions, including residual strains [10,11]. To evaluate DVC performance in terms of acceptable strain errors for a particular tissue and XCT dataset, several tests have been proposed over the years, including virtual displacement of a single tomogram [12], zero-strain test with DVC computed between two consecutive undeformed XCT images [13] and more recently a combination of zero-strain and synthetic deformation of the dataset [14,15]. The necessity of performing such analysis for XCT-based mechanics, particularly when applying DVC to new imaging techniques or materials where limited information is available in the literature, is widely accepted within the DVC community. However, as DVC relies on gray-scale resolvable patterns, soft tissues in their native state are difficult to evaluate due to their low absorption. Visualization of articular cartilage as well as the majority of soft tissues using XCT still relies on the use of radiopaque staining (i.e., iodine potassium iodide, phosphotungstic acid) [16,17], but this type of staining can alter both morphology and mechanical properties of tissues mainly due to dehydration caused by the use of solvents such as ethanol, resulting in tissue shrinkage [18,19,20,21,22,23]. As a solution, in line phase-contrast using high-flux synchrotron radiation (SR-XCT) was used on native articular cartilage [24,25], aiming to enhance image contrast. To date, SR-XCT phase-contrast experiments enabling DVC computation of full-field strain on unstained musculoskeletal soft tissues has only been performed for intervertebral discs [26] and no literature is available on DVC strain changes in articular cartilage. A recent study [27] exploited the natural tissue texture created by hypertrophic chondrocytes in calcified cartilage and osteocytic lacunae in subchondral bone for in situ SR-XCT mechanics to compute load-induced displacements and strains within OA-developing mice knee joints using DVC. However, that study [27] employed only minimal phase-contrast and therefore could not achieve sufficient contrast to obtain full-field strain distribution in the articular cartilage, which suggests how ad-hoc experiments should be tuned to resolve both soft and mineralized tissues simultaneously in order to allow comprehensive DVC analysis of the strain pattern at the cartilage-bone interface.

Setting up experimental protocols to enable feature recognition in soft-hard interfaces using phase-contrast XCT imaging is often constrained by experimental X-ray exposure conditions. Long exposures to high-flux SR-XCT radiation can induce bone microdamage invalidating subsequent strain analysis [28]. To such extent, Barth et al. [29] reported a 21% increase in the concentration of non-enzymatic cross-links in SR-irradiated bone, which in turn restricted fibrillar sliding of the collagen fibrils and lead to a decline in strength, toughness and ductility of bone. The effect of X-ray radiation on articular cartilage degradation has been instead associated with the decline in proteoglycan synthesis, reducing compressive stiffness [30]. Young’s modulus values were also found to be 75% and 60% lower in irradiated articular cartilage when compared to controls using microindentation and nanoindentation, respectively [31]. Lab-based XCT imaging could be a potential solution to reduce X-ray irradiation damage in tissues [32], although the effect of longer acquisition times to enhance phase-contrast is still unexplored. From this perspective, both absorption-based [33] and propagation-based phase-contrast imaging [34] showed great potential to resolve unstained soft tissues including articular cartilage [17,35] in lab-based XCT systems, but applications are still very limited.

Therefore, by further exploring phase-contrast XCT imaging to enhance gray-scale features for subsequent DVC analysis it will be possible, for the first time, to capture the 3D strain changes at the cartilage-bone interface in unstained tissues. The proposed study aims to provide experimental validity on the use of propagation-based phase-contrast XCT imaging procedures in a lab-based system to enable DVC computation in challenging tissue interfaces, such as the cartilage-bone. The suitability of DVC analysis based on phase-contrast XCT images enhancing features of the relatively homogeneous articular cartilage structure, as well as mineralized tissue (calcified cartilage and subchondral bone), will be determined. In the first instance, full-field strain uncertainties and post-mechanics residual strains of the cartilage-bone unit will be evaluated. Additionally, micromechanical probing (indentation) will be used to assess local variations of mechanical properties in the articular cartilage surface due to tissue preparation and X-ray exposure.

## 2. Materials and Methods

**Figure 1:**
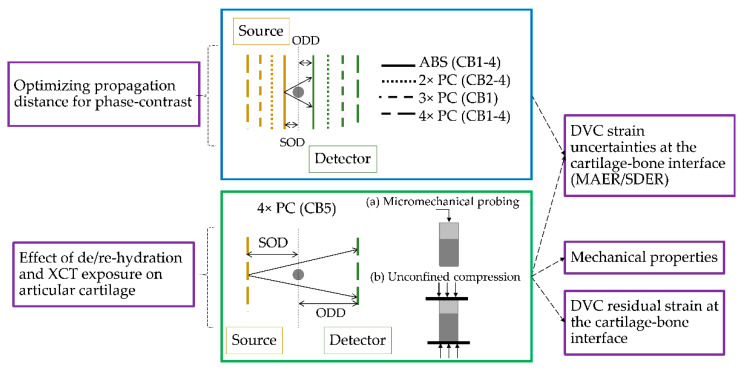
Schematic representation of the two testing protocols used in this study. Specimen CB1 was kept moist during XCT imaging, whereas specimens CB2-5 were maintained fully immersed in Phosphate Buffered Saline (PBS) during image acquisition. Specimens CB1–4 (blue box) were imaged twice to allow for DVC error analysis in absorption and at increased propagation distance up to 4×. Specimen CB5 (green box) was instead imaged only in phase-contrast (4×) twice for DVC error and articular cartilage surface indented (a) to assess effect of de/rehydration and X-ray exposure. The specimen was then mechanically tested under unconfined compression (b) and re-imaged with XCT to compute residual strain via DVC. CB1 and CB5 were also used for synthetic deformation test.

Cartilage-bone bovine plugs underwent two different testing protocols as described in Figure 1. The first explored XCT imaging and the influence of increased propagation distances, ranging from mostly absorption to 4× the effective distance, on DVC correlation ability and strain uncertainties in both articular cartilage and mineralized tissue (calcified cartilage and subchondral bone) at the interface. Based on the propagation data, the second protocol investigated a process of dehydration and rehydration of the tissue in order to enhance XCT contrast (at 4×) by delineating chondrocyte lacunae in articular cartilage [36], while keeping the matrix mechanics unaltered [37]. Micromechanical probing (indentation) of the cartilage articular surface was conducted before, after de/re-hydration and after the first two consecutive XCT tomograms acquired for the DVC zero-strain analysis. Finally, mechanical testing (unconfined compression) was conducted and the cartilage-bone plug re-imaged again with XCT post-mechanics to compute residual strains via DVC.

### 2.1. Specimens

Bovine knee joints were obtained in a local farm (Owtons Chalcroft Farm, Hedge End, Southampton, UK) from healthy adult animals that were sacrificed for alimentary purposes at age between 12 and 20 months. Osteochondral cylindrical plugs of 3 mm (n = 4; namely CB1, CB2, CB3 and CB4) and 4 mm (n = 1; namely CB5) diameter were cored from the medial condyle of the bovine tibiae about an inch from the edge and from the middle section of the intercondylar area where the cartilage surface was the flattest. The bone part of the plug was cut with a scalpel to lengths of 6 mm and 8 mm, respectively leaving the articular cartilage, subchondral bone and part of the underlying trabecular network intact. Fresh specimens were then stored at −20 °C. The 3 mm plugs (CB1-4) were used for strain uncertainty analysis with DVC at different XCT propagation distances and were thawed 30 min before imaging. The 4 mm plug (CB5) underwent a process of dehydration and rehydration adopted from Boettcher et al. [37]. Briefly, the specimen was incubated at room temperature for approximately 36 h and subsequently rehydrated in PBS overnight. The specimen was then mounted on a metal endcap (bone part) before subsequent articular cartilage micromechanical probing (indentation), XCT imaging, mechanical testing and XCT re-imaging.

### 2.2. Micromechanical Probing and Mechanical Testing

FT-RS1002 Microrobotic System with a FT-S10000 Microforce sensing probe (FemtoTools, Buchs Switzerland) was used to record the force and displacement at the flat-tip indentor (flat base area of 50 µm × 50 µm). Micromechanical probing was conducted at room temperature with the specimen fully immersed in PBS throughout the test. Indentations were performed in triplicate at nine different locations (spaced 100 µm apart) across the central area of the articular cartilage surface in the plug and load-control set at 45 mN. The specimen was kept hydrated in Phosphate Buffered Saline (PBS) for 30 min prior to testing and there was a 30 s recovery period between indentations. Force-displacement curves were obtained for fresh articular cartilage, post de/re-hydration and post XCT exposure for DVC zero-strain analysis. The contact stiffness, S = dP/dh, was obtained based on the linear slope of the unloading curve at the initial stage of the unloading from maximum load, where P is the indentation load (N) and h is the indentation depth (µm) [38]. Hardness was also calculated as the quotient of the maximum force over the contact area at each indentation location and test condition [38]. Unconfined compression was then performed on the plug (Bose Electroforce, New Castle, DE, USA) with load applied on the free articular cartilage surface. The specimen was immersed in PBS throughout the duration of the test and pre-loaded with 0.2 N for 3000 s. Compression was then applied as a displacement of 0.2 mm (equal to ~10% of the articular cartilage thickness) at a speed of 0.2 mm/s and held for 120 s to allow stress relaxation [39].

### 2.3. XCT, Image Post-Processing and Synthetic Deformation

Specimens were mounted in sealed polyimide tubes (CB1: in a moist state; CB2-5 immersed in PBS) and imaged in a high-resolution 3D X-ray microscope (Versa 510, Zeiss, Oberkochen, Germany) twice consecutively to allow for DVC zero-strain analysis (except for CB1 where one of the two acquisitions at 4× was unsuccessful). XCT images were acquired (40 keV, 3 W, 3001 projections) in absorption, with both source and detector placed in close proximity to the specimen, and using propagation-based phase-contrast imaging [40] where both the source-to-object (SOD) and object-to-detector (ODD) distance increased by either doubling (2×), tripling (3×) or quadrupling (4×). The exposure time was set such that the detector intensity for the phase-contrast imaging was equal to the absorption detector intensity. Details of imaging conditions for all the specimens are reported in Table 1.

Specimen CB5 was imaged once again at 4× post-mechanics under the same conditions to explore residual strain via DVC. All datasets were reconstructed using standard filtered back projection [41].

Following image reconstruction, the XCT datasets were rigidly aligned (Avizo 9.4, ThermoFisher, Waltham, MA, USA) using as reference the first acquired tomogram. The rigid registration was based on the Normalized Mutual Information optimization metric [42]. The images were then denoized by applying an anisotropic diffusion filter where articular cartilage and mineralized tissue were separately labeled using a watershed segmentation algorithm (Avizo 9.4, ThermoFisher, Waltham, MA, USA). The remaining tissue voxels (i.e., bone marrow) were set to zero grey-scale value [43]. Cubic volumes of interest (VOIs) of variable sizes were cropped from the middle of each image.

Synthetic deformation on specimens CB1 (3× PC) and CB5 (4× PC) was applied using Avizo (v9.4, ThermoFisher, Waltham, MA, USA). The second scan of each specimen was axially compressed (z direction), applying a virtual affine deformation symmetric with respect to the center of the image of 1% (10,000 µε). Lanczos interpolation was then applied to the virtually deformed images.

### 2.4. Digital Volume Correlation

DVC (DaVis v10.05, LaVision GmbH, Göttingen, Germany) analysis was performed to evaluate the 3D full-field strain uncertainties in terms of mean absolute error (MAER) and standard deviation of the error (SDER) [13] using a zero-strain test for all the specimens (CB1−5) and at each imaging condition (abs, 2×, 3× and 4×), where direct correlation was carried out between two consecutively acquired tomograms. In addition, on those samples showing notably enhanced contrast in articular cartilage as well as mineralized tissue (CB1, 3× and CB5, 4×), DVC strain uncertainties (MAER, SDER) and axial strain (*ε_zz_*) were also computed between the first tomogram and the synthetically deformed second one [14]. The DaVis software is based on a local approach of deformable registration and further details on the operating principles of the algorithm are detailed elsewhere [43]. MAER and SDER were computed for both articular cartilage and mineralized tissue at the interface with sub-volumes ranging 16 to 80 voxels, where only the sub-volumes included in the gray-scale of each region were considered. To evaluate the residual strain distribution in the cartilage-bone unit following deformation induced ex situ by the compressive load cycle, the von Mises Equivalent Strain (*ε_eq_*) was computed as in Equations (1)–(5) after performing a multipass DVC with final sub-volume of 48 voxels (~96 µm), reached via predictor sub-volumes of 80-72-64-56 voxels. DVC strain maps were then interpolated onto the 3D image. Similarly, the *ε_eq_* was also calculated from the two zero-strain repeated tomograms to allow for comparison.

(1) $$\varepsilon_{eq}=\frac{2}{3}\sqrt{\frac{3\left(e_{xx}^{2}+e_{yy}^{2}+e_{zz}^{2}\right)}{2}+\frac{3\left(\gamma_{xy}^{2}+\gamma_{xz}^{2}+\gamma_{yz}^{2}\right)}{4}}$$

(2) $$e_{xx}=\frac{2}{3}\varepsilon_{xx}-\frac{1}{3}\varepsilon_{yy}-\frac{1}{3}\varepsilon_{zz}$$

(3) $$e_{yy}=\frac{2}{3}\varepsilon_{yy}-\frac{1}{3}-\varepsilon_{xx}-\frac{1}{3}\varepsilon_{zz}$$

(4) $$e_{zz}=\frac{2}{3}\varepsilon_{zz}-\frac{1}{3}-\varepsilon_{xx}-\frac{1}{3}\varepsilon_{yy}$$

(5) $$\gamma_{ij}=2\varepsilon_{ij}$$

## 3. Results

### 3.1. Phase-Contrast Enhancement and DVC Strain Uncertainties

**Figure 2:**
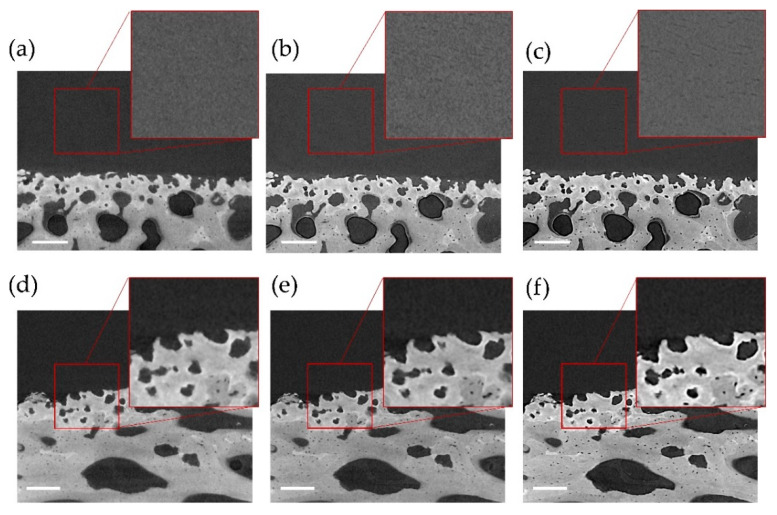
XCT cross-sections of specimens (**a**–**c**) CB1, kept moist throughout imaging and (**d**–**f**) CB4, kept in PBS at increasing propagation distances: (**a**,**d**) absorption, (**e**) 2×, (**b**) 3× and (**c**,**f**) 4× propagation distance for phase-contrast imaging. Details of (**a**,**c**) chondrocyte lacunae and (**d**,**f**) bone-cartilage interface are also shown. Scale bars are 200 µm for all images.

The use of propagation-based phase-contrast imaging enabled the enhancement of both articular cartilage and mineralized features in the tomograms as shown in Figure 2. While a consistent improvement further revealing osteocyte and hypertrophic chondrocyte lacunae was observed in mineralized tissue for all specimens (moist and in PBS), enhancement of chondrocyte organization in articular cartilage was mostly detected with specimen kept moist throughout the duration of the experiment (Figure 2a–c).

**Figure 3:**
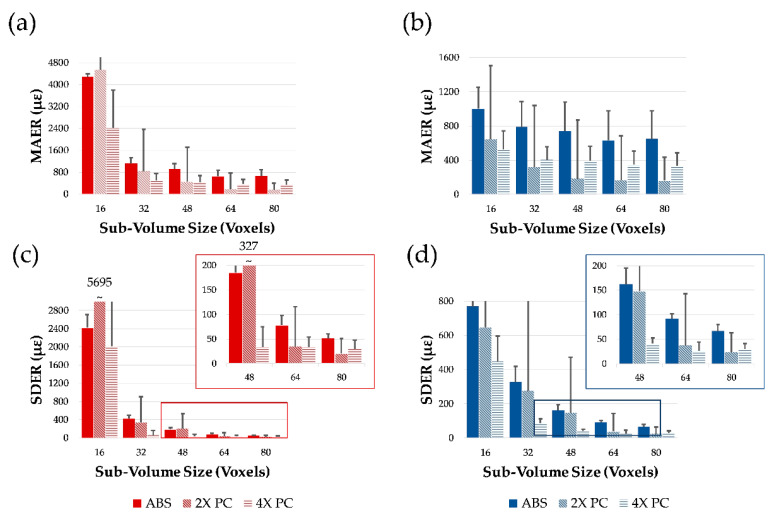
Strain errors of specimens kept in PBS during XCT imaging (CB2–4). (**a**,**b**) Mean absolute error (MAER) and (**c**,**d**) standard deviation of the error (SDER) are shown for (**a**,**c**) the articular cartilage region and (**b**,**d**) the mineralized region of the specimens in absorption (ABS), 2× and 4× propagation-based phase-contrast (PC) at varying sub-volume sizes.

As for plugs immersed in PBS during imaging, both MAER and SDER (median and SD of the three specimens) of the articular cartilage (Figure 3a,c) followed the same typical trend observed in mineralized tissue (Figure 3b,d), in which strain errors decreased with the DVC sub-volume size. 

Also, the effect of phase-contrast (2× and 4×) resulted beneficial in reducing strain uncertainties. When comparing absorption to 4×, MAER in articular cartilage was reduced from 4278 µε to 2417 µε for sub-volume of 16 voxels and from 2418 µε to 343 µε for sub-volume of 80 voxels, whereas in mineralized tissue reduction was from 1002 µε to 522 µε (16 voxels sub-volume) and from 650 µε to 330 µε (80 voxels sub-volume). Similar trends were found for SDER, with strain uncertainties reduced for both articular cartilage (242 0 µε to 2012 µε and 51 µε to 30 µε for sub-volumes of 16 voxels and 80 voxels, respectively) and mineralized tissue (770 µε to 448 µε and 67 µε to 29 µε for sub-volumes of 16 voxels and 80 voxels, respectively). For both regions, the highest inter-specimen variability of MAER and SDER for the PBS cohort was observed for the 2× propagation distance, mostly due to the larger random errors of specimen CB3 both in the articular cartilage and mineralized region (Tables S1 and S2).

**Figure 4:**
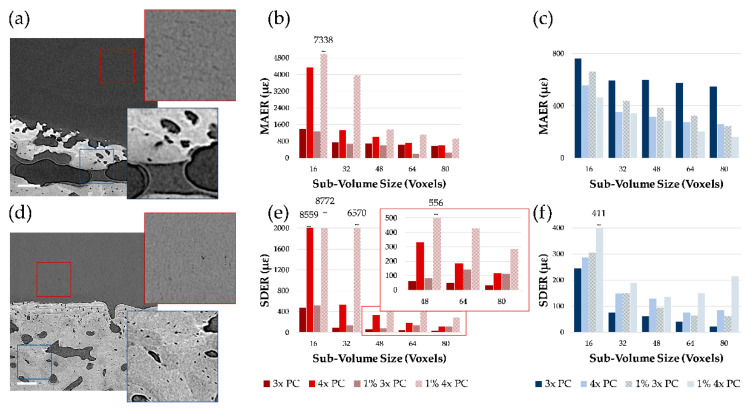
Strain uncertainty analysis for the contrast-enhanced specimens. XCT cross-sections of (**a**) CB1 and (**d**) CB5 samples imaged in propagation-based (3× and 4×) phase-contrast (PC) showing clear features in the articular cartilage region. Scale bars are 200 µm. (**b**,**c**) Mean absolute error (MAER) and (**e**,**f**) standard deviation of the error (SDER) are shown for (**b**,**e**) the articular cartilage and (**c**,**f**) the mineralized regions of the specimens for the zero-strain and 1% synthetic deformation tests at varying sub-volume sizes.

The two specimens that showed clearer features in both articular cartilage and mineralized tissue resulted in CB1 (moist, 3×—Figure 4a) and CB5 (de/re-hydrated, PBS, 4×—Figure 4d). For those, other than calculating MAER and SDER for the zero-strain test, these were also computed between the first tomogram and synthetically deformed (1% compression) second dataset of the pair (Figure 4a,b,d,e). Once more, increasing sub-volume size resulted in reducing MAER/SDER for both regions. CB5 experienced higher uncertainties (zero-strain and synthetic) in the articular cartilage compared to CB1, particularly for smaller sub-volumes (Figure 4b,e). For example, at 48 voxels sub-volume the difference in MAER for the two was approximately 309 µε for zero-strain and 773 µε for synthetic; whereas for SDER it was 271 µε for zero-strain and 474 µε for synthetic. In the mineralized region, an opposite trend was observed for MAER where CB5 performed consistently better than CB1, with differences of approximately 280 µε and 100 µε for zero-strain and synthetic in the 48 voxels sub-volume, respectively. Comparable trends were observed for the random errors of all strain components in both regions (Tables S1 and S2). SDER in the region followed a similar pattern as in the articular cartilage with CB1 performing better than CB5, although in the 48 voxels sub-volume MAER/SDER were <150 µε in all cases.

**Figure 5:**
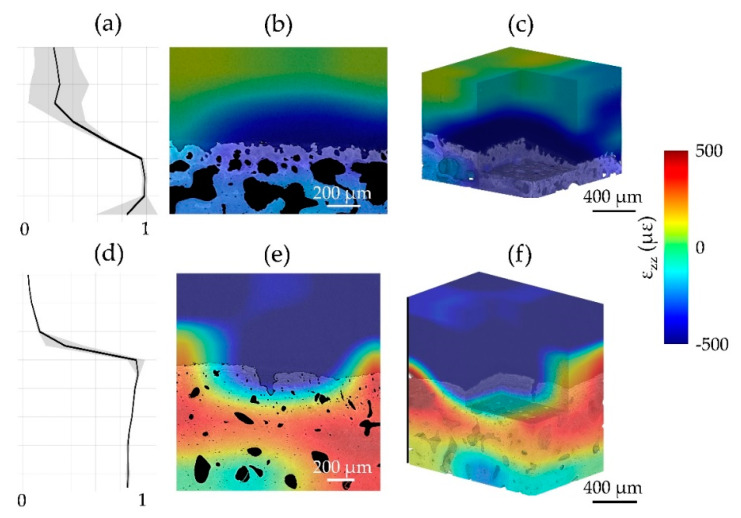
Correlation coefficient and full-field axial strain (*ε_zz_*) representation in zero-strain test for (**a**–**c**) CB1 and (**d**–**f**) CB5 specimens imaged at 3× and 4× propagation-based phase-contrast (PC), respectively. (**a**,**d**) The mean correlation coefficient (black line) across all the slices and its standard deviation (gray shaded region) is shown along the cartilage-bone plug length. *ε_zz_* distribution for a cross-section of (**b**) CB1, (**e**) CB5 and for (**c**,**f**) their volumes were computed using a multipass scheme (48 voxel final sub-volume size).

**Figure 6:**
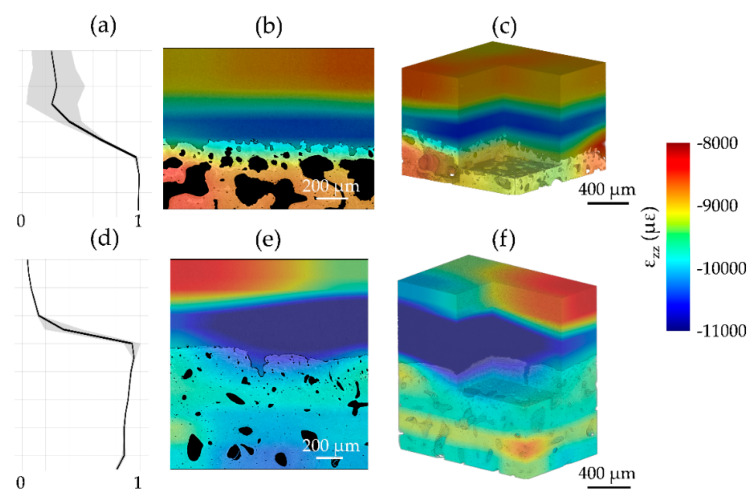
Correlation coefficient and full-field axial strain (*ε_zz_*) representation in synthetic deformation test (1% strain) for (**a**–**c**) CB1 and (**d**–**f**) CB5 specimens imaged at 3× and 4× propagation-based phase-contrast (PC), respectively. (**a**,**d**) The mean correlation coefficient (black line) across all the slices and its standard deviation (gray shaded region) is shown along the cartilage-bone plug length. *ε_zz_* distribution for a cross-section of (**b**) CB1, (**e**) CB5 and for (**c**,**f**) their volumes were computed using a multipass scheme (48 voxel final sub-volume size).

A visual representation of *ε_zz_* at the cartilage-bone interface was computed for both zero-strain (Figure 5) and synthetic deformation (Figure 6), together with the correlation coefficient across articular cartilage and mineralized tissue. It was shown, consistently with MAER/SDER, how the patterns in articular cartilage for CB1 provide a clearer pathway for DVC tracking with lower uncertainty in the zero-strain (Figure 5b,e) and a more consistent deformation distribution in the synthetic case (Figure 6b,e). The enhanced features in CB5 were able to contain zero-strain errors in the tissues (Figure 5e,f) and provided enough gray-scale pattern to localize applied synthetic deformation (Figure 6e,f), despite lower values of the correlation coefficient within the articular cartilage (Figure 5d and Figure 6d) compared to CB1 (Figure 5a and Figure 6a).

### 3.2. Mechanics and Full-Field Residual Strain

**Figure 7:**
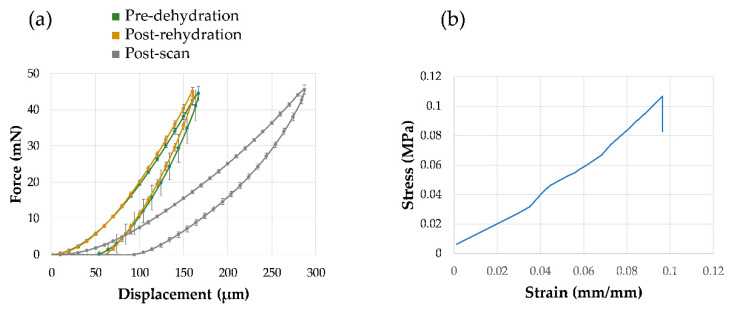
Mechanics of CB5. (**a**) Average force-displacement indentation curves of the cartilage articular surface for fresh tissue, after de/re-hydration and after two consecutive XCT acquisitions at 4× propagation distance. (**b**) Stress-strain response of the cartilage-bone plug after one cycle of unconfined compression applied to the cartilage articular surface (~10% of the articular cartilage thickness).

The process of de/re-hydration of the tissue (CB5), aimed at further enhancing gray-scale features for the specimen immersed in PBS throughout the imaging process, was successful in preserving the articular cartilage micromechanics characterized by microindentation (Figure 7a). However, a long XCT exposure induced a decrease in contact stiffness of ~25% with respect to the fresh tissue as shown in Figure 7a. Hardness was also reduced post-imaging (1.1 ± 0.01 MPa) when compared to the fresh articular cartilage (1.7 ± 0.05 MPa). After XCT imaging for zero-strain analysis, the specimen (CB5) was subjected to unconfined compression applied to the articular cartilage surface (Figure 7b), resulting in a maximum applied stress of ~0.11 MPa and a stress relaxation of ~0.02 MPa.

**Figure 8:**
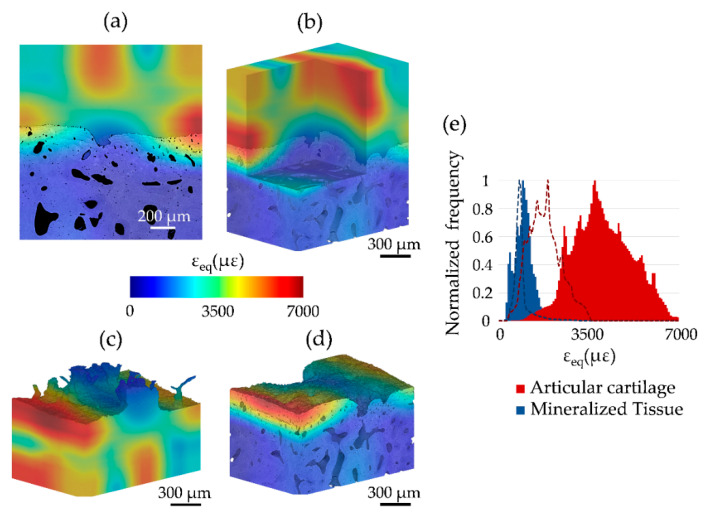
Full-field residual strain distribution computed after mechanical testing (CB5). Equivalent von Mises Strain (*ε_eq_*) distribution for (**a**) a cross-section of the sample, (**b**) the entire volume, (**c**) the articular cartilage and (**d**) mineralized tissue computed using a multipass scheme (48 voxel final sub-volume size). (**e**) Normalized frequency distribution of *ε_eq_* for the articular cartilage and mineralized tissue is also shown (solid region), together with respective distributions in the same tissue regions at zero-strain (dashed lines).

The equivalent von Mises strain (*ε_eq_*) computed post-mechanics showed residual strains mainly localized in regions within the articular cartilage and at the cartilage-bone interface, notably decreasing in the mineralized region toward subchondral bone (Figure 8a,b). Some regions at the interface showed high residual strains (up to ~7000 µε) across both articular cartilage (Figure 8c) and mineralized region, mainly calcified cartilage (Figure 8d). Normalized frequency distribution (Figure 8e) displayed peak *ε_eq_* values of 3710 µε and 910 µε for articular cartilage and mineralized tissue post-mechanics, respectively. Peak values at zero-strain were 1890 µε for articular cartilage and 770 µε for mineralized tissue (Figure 8e).

## 4. Discussion

This study aimed at providing evidence on the use of digital volume correlation (DVC) to measure full-field strain in contrast-enhanced images of unstained cartilage-bone plugs, obtained via propagation-based phase-contrast in lab-based XCT systems. Phase-contrast XCT imaging was explored up to 4× propagation distance and successfully improved contrast in both articular cartilage and mineralized tissue. The transition from absorption to enhanced phase-contrast resulted in improved image texture (Figure 2). As the tissue was only kept moist (CB1) chondrocyte lacunae were clearly visible within the articular cartilage matrix at increased propagation distance (Figure 2c), when compared to specimens fully immersed in PBS (CB2–4, Figure 4f). This may be due to some partial dehydration during prolonged X-ray exposure as well as the relative age of the source animals and the exact location of specimen extraction within the medial condyle, which allowed a more detailed visualization of such features. In fact, more mature articular cartilage presents depth-dependent cell density and organization that can facilitate feature recognition compared to more immature tissue [44]. Other than the articular cartilage, phase-contrast imaging also enhanced mineralized regions at the interface with hypertrophic chondrocyte and osteocyte lacunae, most importantly, preserving the gray-scale quality at the cartilage-bone interface. This is vital, as previous phase-contrast SR-XCT on cartilage-bone plugs was successful in visualizing structural details in the articular cartilage (chondrocyte distribution) [25,45], although typical refraction brightness was visible in the articular cartilage matrix and entirely covered mineralized microarchitecture [25]. Such image gray-scale alteration and noise due to the use of phase-contrast may affect characterization [24], particularly at the cartilage-bone interface.

The overall quality of the tomograms enabled DVC computation in both regions at the interface. As in any new application, DVC strain uncertainty tests such as zero-strain [13] and synthetic deformation [14,15] is required. This is of paramount importance to evaluate appropriate DVC settings that would ensure a more reliable measurement of the full-field strain developed under applied (in situ) or previous (ex situ) loading. The results in this paper (Figure 3 and Figure 4) are in line with typical trends observed in previous DVC analysis of bone and bone-biomaterial systems [13], where errors decreased with increasing sub-volume size. However, some peculiarities in the behavior were found in this study, such as large variability in MAER/SDER for both articular cartilage and mineralized tissue (specimens in PBS) at 2× propagation distance (Figure 3a–d). This is interesting as it suggests how intermediate feature unraveling with phase-contrast may be interpreted as noise in DVC, but this becomes clearer at 4× propagation distance where the contrast is sufficient to resolve such features properly over the noise. Another interesting observation is in relation to the synthetic deformation test for two of the specimens (CB1 and CB5). Better feature quality enabled zero-strain and synthetic tests to be comparable (mostly within the same order of magnitude) at intermediate sub-volumes (i.e., 48 voxels and 64 voxels). This is particularly evident for articular cartilage in CB1 (Figure 4b,e) and mineralized tissue in CB5 (Figure 4c,f). Lower contrast or insufficient feature density in the sub-volume may have triggered some difficulties in capturing synthetic strain from DVC, as shown for the articular cartilage region in CB5 (Figure 4b,e). Synthetic strain uncertainty was consistently higher than the relative zero-strain counterpart for all the cases except for MAER with mineralized tissue in CB1, where phase-contrast feature enhancement could lead to unraveling. Overall, the synthetic vs zero-strain results from this paper are in line with previous studies performing synthetic deformation analysis with DVC in cortical bone [14,15]. Also, the visual mapping of *ε_zz_* at the cartilage-bone interface (Figure 5 and Figure 6) reinforces such consideration. Despite the correlation coefficient decreasing in the articular cartilage toward the surface, synthetic deformation had enough gray-scale features to capture deformation imposed at the interface, more clearly for CB1 (Figure 5c) when compared to CB5 (Figure 6c).

The treatment of de/re-hydration of the tissues was based on the hypothesis of partially clearing chondrocyte lacunae during dehydration [36] and then restoring properties of cartilage matrix via subsequent rehydration [37], to potentially enhance feature recognition in phase-contrast XCT. Micromechanical probing (indentation) results (Figure 7a) showed how the treatment was successful in restoring overall articular cartilage mechanical properties, in line with findings reported by Boettcher et al. [37]. Interestingly, prolonged X-ray exposure had a larger effect in reducing stiffness and hardness of the articular cartilage. This is not surprising as exposure to X-ray radiation has already shown to degrade mechanical properties both in bone [11,28] and articular cartilage [30], with different degenerative mechanisms. Bone deterioration can be attributed to an increase in collagen cross-linking with subsequent increase in brittleness and stiffness [29], whereas articular cartilage is thought to be more affected by a reduction in stiffness associated with proteoglycan degradation [30] that is consistent with the results of this study. This is very important information for further research involving propagation-based phase-contrast in both lab and SR-XCT. Recently, Clark et al. [17] reported a protocol combining articular cartilage staining and phase-contrast XCT to resolve chondrocyte feature, potentially aiming at a future use of DVC. However, as staining of soft tissues has been shown to modify morphology and mechanical properties [18], long exposure due to phase-contrast imaging may effect these changes more in stained specimens.

Other than evaluating DVC strain uncertainties, this study aimed at exploring residual strain accumulated post-mechanics at the cartilage-bone interface. CB5 was subjected to unconfined compression adopting a testing protocol reported in [46] (Figure 7b). Residual strain maps show distribution of equivalent von Mises strain (*ε_eq_*) across the interface (Figure 8). Residual strain not only accumulated in the articular cartilage (Figure 8b,c) but also in calcified cartilage and subchondral bone at the interface (Figure 8a,d), suggesting an extremely optimized load-transfer mechanism despite the small applied stress (~0.11 MPa) during the single load cycle. This is also confirmed by the normalized strain frequency distribution in the two regions (Figure 8e) showing how mechanical transfer took place when compared to the zero-strain analysis. The high local values (up to ~7000 µε) at the cartilage-bone interface may be somehow amplified due partial border effect [47], or poorer correlation when extending into cartilage. However, considering that swelling-induced residual compressive strains in the deep zone of articular cartilage can range between ~5000 µε and ~10,000 µε [48], the current results may be real and consistent. Thus, residual strains measured in the mineralized region at the interface are very interesting. It can be speculated that compliance in the cartilage-bone unit has the ability to transfer high local strain (i.e., >10,000 µε for bone yielding [49]) to the calcified cartilage, which in turn ‘modulates’ strain levels transferred to subchondral bone; hence, controlling the remodeling process. This claim is supported by a recent study [27], in which in situ SR-XCT mechanics and DVC of mice knee joints before and after development of OA suggested how in healthy mice high compressive strains (up to ~9000 µε) were mainly localized in the calcified cartilage and then shifted toward subchondral bone during the onset of OA when the calcified cartilage morphology was notably altered, which could subsequently trigger subchondral plate thickening and (less compliant) cartilage damage in OA. However, in that study [27] strain measurement was only conducted in mineralized regions (calcified cartilage and subchondral bone), where the articular cartilage contribution could not be investigated. The current study only evaluates residual strains at the cartilage-bone interface in healthy specimens and further work is needed to better understand the load-transfer ability in both the healthy and diseased joints.

This study has further limitations. First of all, there was a limited number of specimens (n = 5) and imaging conditions (i.e., a few selected steps of phase-contrast). The number of experiments and the way mechanical properties were investigated (i.e., micromechanical probing and testing only for one specimen) are making the study unable to support statistical analysis. Low numerosity is a typical drawback of XCT-based experiments for DVC, which are very time consuming (the current one in particular for longer acquisition times enabling phase-contrast imaging) and ultimately aim at providing a more qualitative, rather than quantitative evaluation. In addition, DVC analysis was only conducted to compute full-field strain uncertainties (MAER/SDER) and residuals post-mechanics to understand how this technique could measure small strain changes at the cartilage-bone interface with gray-scale features enhanced by phase-contrast imaging. Overall, these findings highlight the need for careful planning further research using propagation-based phase-contrast XCT enabling DVC analysis to evaluate cartilage-bone mechanics in both unstained and stained tissues. Limiting X-ray exposure by employing new generation staining agents with potential of better preserving tissue integrity [18], as well as enabling faster phase-contrast retrieval [34] should be further explored to define the right trade-off between tissue integrity and reliability of DVC strain measurements; ultimately, paving the way to a deeper understanding of the cartilage-bone system.

## 5. Conclusions

This study aimed at exploring the use of propagation-based phase-contrast XCT imaging (up to 4× propagation distance) with specimens in different conditions (moist, in PBS and in PBS after treatment of de/re-hydration) to enable DVC analysis at the cartilage-bone interface, including articular cartilage and mineralized tissue (calcified cartilage and subchondral bone). Full-field strain uncertainties for DVC in both articular cartilage and mineralized tissue were computed in the form of MAER/SDER for zero-strain and synthetic deformation tests. The results show how strain error decreased with larger sub-volume size and this was further reduced using phase-contrast for all cases, with synthetic deformation generally experiencing higher strain uncertainties than zero-strain. Enhancement achieved with phase-contrast enabled zero-strain, synthetic deformation and allowed determination of residual strains in the tissue post-mechanics. Local residual strains up to ~7000 µε were found in articular cartilage directed toward mineralized tissue, suggesting high compliance of the cartilage-bone system. To assess changes in articular cartilage properties due to treatment of de/re-hydration and X-ray exposure at 4× propagation distance, micromechanical probing (indentation) of the articular surface was performed. Decrease in both stiffness and hardness of the irradiated articular cartilage were observed, highlighting the importance of controlling phase-contrast settings to achieve an optimal trade-off between tissue integrity and DVC measurement. The findings of this paper will be pivotal for further development of contrast-enhanced DVC with phase-contrast, leading the way to a deeper understanding of the cartilage-bone mechanics.

## Figures and Tables

**Table 1:** Summary of source-to-object distance (SOD), object-to-detector distance (ODD), voxel size and exposure time for all specimens.

Specimen	Position	SOD (mm)	ODD (mm)	Voxel Size (µm)	Exposure Time (s)
CB1	ABS	8.5	20	2.02	2
	3×	25.6	60	2.02	20
	4×	34.2	80	2.02	30
CB2-4	ABS	8.5	20	2.56	2
	2×	17	40	2.56	7.5
	4×	34.1	80	2.56	30
CB5 ^a^	4×	44.4	104	2.03	35

^a^ SOD and ODD in 4× position were determined by 1) placing source and detector in close proximity to the specimen and 2) quadrupling the SOD and ODD.

## Supplementary Materials

The following are available online at https://www.mdpi.com/1996-1944/13/11/2579/s1, Table S1: Random errors of each strain component in the articular cartilage region, Table S2: Random errors of each strain component in the mineralized region.
