# Supplementary material for: Full-Field Strain Uncertainties and Residuals at the Cartilage-Bone Interface in Unstained Tissues Using Propagation-Based Phase-Contrast XCT and Digital Volume Correlation
Source: Materials (Basel). 2020 Jun 5;13(11):2579. doi: 10.3390/ma13112579 (PMC7321571; doi:10.3390/ma13112579)
Supplement: Supplementary file 1 [file materials-13-02579-s001.pdf]

materials

Supplementary Materials

Full-Field Strain Uncertainties and Residuals at the Cartilage-Bone Interface in Unstained Tissues Using Propagation-Based Phase-Contrast XCT and Digital Volume Correlation

Gianluca Tozzi [1,*], Marta Peña Fernández [1,2], Sarah Davis [3], Aikaterina Karali [1], Alexander Peter Kao [1] and Gordon Blunn [3]

[1] Zeiss Global Centre, School of Mechanical and Design Engineering, University of Portsmouth, Portsmouth PO1 3DJ, UK; martapf@kth.se (M.P.F.); katerina.karali@port.ac.uk (A.K.); alex.kao.port@gmail.com (A.P.K.)
[2] School of Engineering Sciences, KTH Royal Institute of Technology, 100 44 Stockholm, Sweden
[3] School of Pharmacy and Biomedical Science, University of Portsmouth, Portsmouth PO1 2DT, UK; sarah.davis@port.ac.uk (S.D.); gordon.blunn@port.ac.uk (G.B.)
* Correspondence: gianluca.tozzi@port.ac.uk; Tel.: +44-23-9284-2514

Table S1. Random errors of each strain component in the articular cartilage region for all the specimens imaged in absorption (ABS), 2×, 3× and/or 4× propagation-based phase-contrast. Values are computed using digital volume correlation (DVC) with a final sub-volume of 48 voxels (~96 μm).

Specimen	Position	Strain Random Errors (με)					
		ε_{xx}	ε_{yy}	ε_{zz}	ε_{xy}	ε_{xz}	ε_{yz}
**CB1**	ABS	634	347	560	295	286	316
	3×	301	194	212	136	144	131
	1%—3×	276	187	607	128	158	163
CB2	ABS	626	320	373	380	553	347
	2×	595	466	212	199	457	174
	4×	114	73	82	48	81	34
CB3	ABS	375	208	819	264	392	510
	2×	1353	1954	954	1371	665	1236
	4x	470	210	403	232	281	300
CB4	ABS	347	396	651	149	268	322
	2×	331	136	391	169	141	157
	4×	140	115	137	61	118	85
CB5	4×	623	951	315	489	383	810
	1%—4×	705	1782	1298	376	419	1278

Table S2. Random errors of each strain component in the mineralized region for all specimens imaged in absorption (ABS), 2×, 3× and/or 4× propagation-based phase-contrast. Values are computed using digital volume correlation (DVC) with a final sub-volume of 48 voxels (~96 μm).

Specimen	Position	Strain Random Errors (με)					
		ε_{xx}	ε_{yy}	ε_{zz}	ε_{xy}	ε_{xz}	ε_{yz}
**CB1**	ABS	239	135	269	97	109	237
	3×	206	91	158	92	110	106
	1%—3×	215	102	412	98	130	146
CB2	ABS	171	219	523	132	248	180
	2×	288	233	380	70	338	193
	4×	48	60	137	39	50	38
CB3	ABS	194	116	963	79	172	236
	2×	657	1241	951	519	697	1863
	4×	139	94	150	74	75	94
CB4	ABS	222	170	521	69	185	187
	2×	68	78	292	57	81	123
	4×	150	82	105	36	52	96
CB5	4×	153	300	242	69	190	455
	1%—4×	173	331	293	86	234	499

© 2020 by the authors. Licensee MDPI, Basel, Switzerland. This article is an open access article distributed under the terms and conditions of the Creative Commons Attribution (CC BY) license (http://creativecommons.org/licenses/by/4.0/).
